# Rethinking Schizophrenia: Beyond the Voices of Schizophrenia

**DOI:** 10.1192/j.eurpsy.2024.1585

**Published:** 2024-08-27

**Authors:** D. Suchonova, P. Kéri

**Affiliations:** ^1^European Brain Council; ^2^GAMIAN-Europe, Brussels, Belgium

## Abstract

**Introduction:**

Despite improvements and innovation in recent years, people living with schizophrenia face variations in access to optimal treatment and care. There is a lot about schizophrenia that is not fully understood, and the high-quality care and support needed by people living with this condition is often unavailable.

**Objectives:**

Develop an evidence-based, compelling policy narrative on schizophrenia Engage a pan-European, multidisciplinary group of experts Offer concrete tools to patient and professional advocacy groups Disseminate findings Draw from our findings practical solutions on how to implement recommendations

**Methods:**

Based on carefully selected existing literature and available resources, **literature review** includes but is not limited to: Value of Treatment Recommendations, Global Burden of Disease Study, Comprehensive Mental Health Action Plan, Mental Health Atlas. The aim is to establish state of play, identify problems and solutions and take stock of current recommendations.

We established a **multi-disciplinary working group** to lead the project, and ensured that representation on this group is cross-disciplinary and cross-sector. The expert group includes country-level patient advocates and clinical leads including key opinion leaders (KOLs) to keep the project focused on what is happening at a national level, and to help create ownership at the national level to take recommendations forward within each country.

We conducted **qualitative semi-structured interviews** with people living with schizophrenia where they provided their insights into how to rethink the way we deal with schizophrenia.

**Results:**

*Provide clear, concrete and adaptable solutions Joint ownership by key stakeholder groups of a common policy narrative on schizophrenia Sustained policy engagement on schizophrenia at the EU and national level*

**Conclusions:**

There is a clear need to rethink the management of schizophrenia and redesign the care pathways to ensure optimal treatment and care for all people living with schizophrenia in Europe. Based around patient testimonies, the aim of the session is to highlight the need to optimise the way we manage schizophrenia by building a strong, coherent, evidence-based policy narrative which speaks to the current priorities in schizophrenia and draws from the current policy landscape in Europe.

Experts involved in the *Rethinking Schizophrenia* project, coordinated by the European Brain Council, have explored the ways in which we can and need to change the way we deal with schizophrenia. The *Rethinking Schizophrenia* project falls under the *Rethinking the management of brain disorders* series, research-driven projects offering policy recommendations to make tangible changes with the aim to improve the lives of people living with brain disorders, neurological and mental alike, across Europe.

**Disclosure of Interest:**

None Declared

